# Helping to heal nature and ourselves through human-rights-based and gender-responsive One Health

**DOI:** 10.1186/s42522-020-00029-0

**Published:** 2020-11-16

**Authors:** Julie Garnier, Sara Savic, Elena Boriani, Brigitte Bagnol, Barbara Häsler, Richard Kock

**Affiliations:** 1 Odyssey Conservation Trust, Bakewell, Derbyshire, DE45 1LA England; 2 https://www.ecohealthinternational.org/regional-chapters/europe/; 3 Naucni institut za veterinarstvo “Novi Sad”, Scientific Veterinary Institute “Novi Sad”, Rumenacki put 20, Novi Sad, 21000 Serbia; 4 Department of Anthropology, University of the Witwatersrand, Johannesburg, South Africa; 5 Department of Pathobiology and Population Sciences, Royal Veterinary College, Hawkshead Lane, North Mymms, Hatfield, Hertfordshire, AL9 7TA UK

**Keywords:** One Health, Gender, Nature, Biodiversity, Indigenous, Health, Women, Climate change, Human rights, COVID-19, Resilience

## Abstract

The health of our planet and humanity is threatened by biodiversity loss, disease and climate crises that are unprecedented in human history, driven by our insatiable consumption and unsustainable production patterns, particularly food systems. The One Health approach is a pathway to synergistically addressing outcomes in term of health and sustainability, but gender issues at the One Health and biodiversity nexus are largely ignored.

By examining the roles and responsibilities of Indigenous and Local People, and especially women, in conserving natural resources, and the social costs of living at the Human-Animal-Environment interface under current conservation strategies, we show that women bear a disproportionate health, poverty and climate burden, despite having pivotal roles in conserving biodiversity. To mitigate risks of emerging infectious diseases, food insecurity and climate change impacts, a gender perspective has previously been proposed, but implementation lags behind. Endemic zoonotic diseases, human-wildlife conflict and environmental pollution lack gender-sensitive frameworks. We demonstrate that women can be powerful agents for change at all levels of society, from communities to businesses, and policy-making institutions, but gender inequalities still persist.

We develop a framework for mainstreaming a gender-responsive and rights-based One Health approach, in order to heal ourselves and nature. Using a leverage-points perspective, we suggest a change of paradigm, from the pursuit of GDP and over-consumption, to a focus on human well-being and their reconnection with healthy environments, using a One Health understanding of nature and health. We recommend learning from Indigenous People to re-position ourselves within nature and to better conserve biodiversity. We also propose integration of gender equity in leadership, the respect of human rights, women’s rights (access to health care, healthy food, land tenure, natural resources, education, and economic opportunities), and the rights of nature, through the implementation of gender-responsive and rights-based One Health Action Plans, at policy-making level, in the private sector and the civil society. As the COVID-19 pandemic continues to unveil deep socio-economic inequities in the wealthiest economies and the vital role of nature in supporting our health, we argue to seize this opportunity to build back better and improve resilience and sustainability by using a gender-responsive and rights-based One Health approach.

## Background

### A biodiversity crisis we failed to address

“Nature is angry. And we fool ourselves if we think we can fool nature. Because nature always strikes back. And around the world, nature is striking back with fury”, stated A. Guterrez at the 2019 Climate Action Summit [1], four months before the global SARS-CoV-2 pandemic [2]. The interrelated biodiversity, health and climate crises that we face are unprecedented in human history, and the health of our planet and our future have never been so threatened. Biodiversity, the very basis of ecosystem functioning and resilience, is currently being lost at a magnitude that threatens Earth‘s ability to sustain future human life [3–5]. Scientists across disciplines agree that humanity is on an environmental precipice. The disintegration of the planet’s life-supporting functions is now more obvious to the world, as climate change and the destruction of some of the most emblematic ecosystems impacts the world’s population and global health, triggering the declaration of a planet emergency crisis [6], the magnitude of which has been fully revealed by the SARS-CoV-2 pandemic.

Exponential increases in resource extraction, combined with intensive production patterns and unsustainably high consumption levels, have resulted in massive land degradation, biodiversity loss, overexploitation and stock depletion of natural resources, pollution, emergence of infectious and the surge in non-communicable diseases, and climate change [4, 7]. Intensification of food production systems, associated with the “Green Revolution’s” aim of preventing widespread food insecurity, has lifted millions of people out of poverty [8, 9]. However, land conversion for agriculture is now known to have the highest relative impact on ecosystem degradation compared to other drivers, which include exploitation of organisms, climate change, pollution and invasive alien species [10]. Since these drivers result from a number of often interconnected underlying factors, which may be demographic, sociocultural, economic, technological, political or institutional, they can lead to unintended consequences, especially on nature, gender and human rights.

Whether marine or terrestrial, natural systems are being destroyed, converted or manipulated to accommodate the insatiable demand for infrastructure, services, food, energy and profit that relate to the economic growth and consumerism of growing populations [11]. World population growth predicted to reach nearly 10 billion by 2050, will inevitably drive further extraction of natural resources from the remaining biodiverse landscapes, in order to satisfy developing economies and sustain old ones [12].

More than one third of the Earth’s land surface is now devoted to croplands and grazing lands, and as a consequence half of the world’s temperate and tropical forests, home to 80% of the world’s terrestrial biodiversity, have been cleared [4]. Recent fires in the Amazon and on other continents threaten to trigger a tipping point, leading to irreversible changes in regional and global weather patterns, consequently depriving a million indigenous people of their homes and livelihoods. Since the start of the modern era, 87% of wetlands, which act as a greenhouse gases regulator and a natural buffer against extreme weather events for millions of people, have been lost through irreversible conversion [4]. Similarly, drylands, which make up more than 40% of the Earth’s surface, are increasingly being cultivated, thereby accelerating desertification and leading to extreme water stress, which now affects a quarter of the world population, and drives political instability and migration [13].

By damming two thirds of the world’s rivers, mainly to irrigate non-locally adapted crop varieties and to produce energy exacerbated by overexploitation, we have lost most freshwater fish populations [4]. Similarly, having depleted the oceans’ fish stocks and exploited more than 90% of the world’s fisheries beyond sustainability, oceans are now perceived as emunctory organs that are suffering from deoxygenation, nutrient loading and massive plastic pollution, at all trophic levels, as well as the effects of climate change [14, 15]. The rapid and massive expansion of hidden “dead zones” and of floating garbage islands throughout the oceans, and the detection of microplastics in several species across the marine food web, including ourselves, are symptoms of the insatiable consumptive lifestyles in the most affluent as well as developing economies [16, 17].

At a species level, around one million animal and plant species are now threatened with extinction and the sixth mass extinction is considered to have started [18]. Extinction rates are about a thousand times those of pre-human times and populations of land vertebrates have declined of 60% between 1970 and 2014, across all countries of the globe, irrespective of national income or status of socioeconomic development [18].

By failing to acknowledge our interconnectivity with the natural world, and how vital biodiversity is to life, we lose perspective on the changes in microbial biodiversity and emerging pathogens that can ultimately lead to a global health crisis. The COVID-19 pandemic is an ultimate warning signal to humanity that we need to reposition ourselves within nature and to adopt a much humbler and caring attitude. Efforts to reconnect with nature have been largely insufficient, partly due to the increased complexity in global resource systems, which limits people’s understanding of the impacts of their activities on nature [19]. These efforts are also hampered by the processes of urbanization and reduced access to green spaces, as well as an increased use of digital connectedness and artificial intelligence, which reinforces humans’ anthropocentric view of life. However, with the COVID-19 pandemic and the indisputable evidence that our unsustainable consumption and production patterns are key drivers of biodiversity loss and disease emergence, it is now clear that we cannot continue “business as usual” [4].

### An old development paradigm drove our ill-health

The requirement to feed a growing global population and a rising demand for animal source foods, are reasons still used to promote and defend the clearing of massive land areas for intensive agriculture, which is one of the main drivers of biodiversity loss and emergence of new infectious diseases [4, 20]. Today’s food systems are unbalanced, as they fail to address the double burden of under- and overnutrition in many populations, where women carry most of the health and poverty burden [21]. Moving from a natural food system based diet, using a diversity of seasonally available foodstuffs, towards the modern diet, consuming manufactured highly processed, high-density, high sugar content, and homogenized food, that rely on three staple crops (rice, wheat and maize), has contributed substantially to non-communicable diseases (NCDs) prevalence and global health challenges [22]. Globally, 39% of the world population is overweight with steep trends among adults and school-age children, increasing their risks of cancer, diabetes mellitus and cardio-vascular diseases making an unhealthy diet the leading risk factor for deaths worldwide [23]. Such underlying health conditions, like hypertension or diabetes mellitus, now also appear to be associated with a more severe progression of the COVID-19 disease in infected patients [24].

Simultaneously, hunger is increasing again after a decade-long decline, affecting 820 million people, with slightly higher prevalence rates across all continents in women than in men [23]. Gender-based differences in communities of low socioeconomic status, associated with poor nutrition and low education levels, contribute to maintain poverty cycles, in which children fail to reach their genetic potential. The cumulative negative effects across generations lead to a vicious spiral of increasing health problems and diminishing productivity [21]. Girls affected by poor growth, both in foetal and early life, are more likely to give birth to low-birthweight babies, thus projecting poor nutrition and increased risk of NCDs to the next generation [25]. Similarly, but at the opposite extreme, obesity in women can be perpetuated into future generations, since being overweight during pregnancy increases the risk of the child becoming obese or overweight later in life [26]. A gender-sensitive, systems approach is urgently required to address effectively the growing problem of malnutrition and NCDs [23].

Externalities associated with fossil fuel based energy production represents one of the greatest environmental health risks. Air pollution is associated with more than seven million deaths worldwide and plays a role in increasing the risks of stroke, diabetes, lung cancer and chronic lung diseases [27]. As with most other environmental health risks, the burden falls hardest on the most vulnerable and exposed in societies, including women and children for whom such health challenges are a matter of life and death [28]. Indoor pollution from the burning of solid fuel, which is still used by 3 billion people, is the greatest silent killer of women and girls in Low-to-Middle-Income Countries (LMICs), where gender roles dictate that women and girls prepare meals in poorly ventilated kitchens (more than 60% of the 3.8 million premature deaths from household air pollution are among women and children) [29]. Women’s status was identified to be a robust predictor of solid fuel use, and improved women’s status also correlated directly with lower female to male indoor air pollution deaths ratios, and indirectly with reduced female death prevalence through lower solid fuel dependence [28].

Animal and plant diseases have also evolved at increasing speed in domestic animal breeds and crop varieties of limited genetic diversity, which are industrially produced and managed at densities requiring continuous use of vaccines and pharmaceuticals in livestock and the frequent application of pesticides on crops. Consequently, environmental pollution and drug-resistant pathogens have become an integral part of the risk frame of industrialized food systems [30]. Health gains of more than a hundred years in drugs and anti-microbial development are now in jeopardy with a current burden of Antimicrobial Resistance (AMR) estimated at 700,000 deaths annually and a projected one of 10 million deaths by 2050 [31, 32]. Disadvantaged populations and especially women and children living in remote rural areas, with a lack of adequate water, sanitation facilities and appropriate healthcare, may face greater exposure to pathogens and increased vulnerability if they contract an antibiotic resistant infection [33].

### Integrated One Health sciences and policies

Understanding that the health of people is inevitably linked to the health of ecosystems is not a new concept. The holistic healing practitioners in China and India propagated a healthy way of living in harmony with nature as far back as 5000 years ago. For the last 20 years, the fields of Ecohealth, One Health, and Planetary Health, have (re-)emerged and formally acknowledged the need to recognize that human, animal and environmental health are inextricably connected.

One Health and Ecohealth converge in promoting a paradigm shift towards a system approach with a focus of restoring resilience of biological systems at all scales [34]. The Planetary Health approach promotes the development of integrated policy solutions that address environmental sustainability together with human health [11]. These new fields have allowed the gathering of scientific evidence on how humans have contributed to the ill-state of our planet and how the health of ecosystems and the diversity of nature is foundational to our health [11, 19]. Such system thinking will help in rebuilding a jigsaw of disintegrated pieces for which we are still missing many of the connections, as further complex ecological processes and interactions that sustain all life forms are still to be discovered.

Implementing integrated health approaches delivers added value and multiple outcomes in term of sustainability, health, welfare, equity, and effectiveness [35]. Given the diversity of One Health initiatives, there is no single outcome that can summarize One Health endeavors but rather a wide range of different outcomes and impacts, expected as well as unexpected, that include disciplinary, interdisciplinary and One Health outcomes and impacts [35]. The framework developed by the EU COST Action “Network for Evaluation of One Health”, now Network for Ecohealth and One Health, allows for capturing such impacts and evaluating One Health initiatives.

There are case studies of using a One Health approach to manage successfully endemic zoonotic diseases [36, 37]. A One Health approach also proved critical in improving understanding of the origin and drivers of the emergence of diseases such as SARS, MERS and in helping to contain the outbreaks efficiently [38]. The PREDICT project initiated in 2009 also made very significant contributions to understand emerging viral threats and to strengthen global surveillance of such threats [39]. In the context of the global public health emergency caused by the COVID-19 pandemic, the One Health approach is now being recognized as the way forward to address current and future emerging pathogens threats [37, 38].

On the policy side, the creation of instruments aimed at guiding development such as the 2030 Agenda for Sustainable Development has enabled pathways to address the deep systemic changes required. By reflecting a wide range of environmental, societal and economic concerns that are all inter-linked and embedded into each other, the 2030 Agenda proposes a holistic approach, although some of the Sustainable Development Goals (SDGs) are antagonistic to each other in the context of the current political economies [34]. This is an irony, but symptomatic of the tensions in growth-based economies, and reflective of the superficial interest in nature, which persists in global communities. The SDGs show a strong commitment towards sustainability, but effective progress made towards achieving the 2030 Agenda is slower than needed in many areas to meet the targets, largely because the lack of empowerment of the most vulnerable groups including women [40].

The gender dimension of the 2030 Agenda has now been fully recognized and gender equality has been identified as fundamental to delivering the SDGs and mitigating climate change [41, 42]. The need to take a gender and equity focus in efforts to protect and improve population health is also mentioned in a variety of mandates and instruments, including WHO’s most recent 13th General Programme of Work (2019–2023) [43]. In the current COVID-19 pandemic context, there has also been a call to address gender and health inequities, in order to respond to the outbreak effectively [44].

In the One Health policy domain, the gender dimension is only succinctly recognized as being an important cross-cutting dimension in operationalizing One Health, with the need to consider gender in developing communication strategies and in the development, implementation and evaluation of country plans [45]. Some One Health networks, such as Africa One Health University Network (AFROHUN), recognize the role and importance of addressing gender issues in One Health. In response, they have developed a Gender, One Health and Infectious Disease short course to apply gender analysis tools to disease surveillance, response, and control [46]. The relevance of integrating a gender dimension in Ecohealth and of using gender analysis tools in a One Health approach to infectious disease surveillance, response and control, have also been suggested [47, 48]. In reality, the One Health/Ecohealth community, in addition to those in conservation and development, are still failing to consider the critical importance of adopting and implementing a gender-sensitive One Health approach, not only to respect a human-rights dimension but also to bring transformative change in finding new sustainable pathways to the environmental, health and climate crisis that we are facing. In one of the most recent institutional frameworks proposed to fundamentally change the agricultural landscape in the context of SDG2, there is not even a single mention of gender issues [49].

This paper originates from work conducted by the main authors, J. Garnier and R. Kock, in the context of biodiversity and wildlife conservation and One Health projects in Africa mainly and further developed in the context of NEOH (Network for Ecohealth and One Health; previously called Network for Evaluation of One Health). We first describe some of the social costs borne by Indigenous People and Local Communities living in biodiverse areas and around protected areas, as well as their relationship to nature, before reviewing the main roles and responsibilities that women in these communities have in managing natural resources, which exposes them to an increased health burden, but make them play a critical role in conserving biodiversity. We then suggest a set of recommendations for mainstreaming a One Health approach, based on the respect of human rights, the rights of nature and gender equity at all levels, from policy-making institutions to private sector and civil society, embedded in a framework whose ultimate goal aim is to reposition ourselves within nature to care and protect it and ultimately improve well-being for all.

## A proposal for human-rights and gender-responsive One Health

Supporting evidence is summarized in Table 1.

**Table 1:** Summary of challenges, risks and gender roles that were examined at the interface Human / Animal / Wildlife, with some known gender-sensitive and rights-based measures and their known outcomes

Challenges /Risks at the interface	Characteristics	References
Emerging Infectious Disease	Increased risks in regions with higher human densities and fragmented habitats, wildlife markets. Identification of sex-based (e.g. pregnancy) and gender-based differences in risks and exposure between men and women (e.g. hunters, women with wild meat preparation, women as family health carers etc). Gender Analysis conducted for some EIDs (Ebola, Zika, HIV) & Training initiated. Needs integration of environmental component and implementation.	[37, 47, 50–57]
Endemic Zoonotic Diseases	Increased risk in low resource settings. Risks associated with gender roles have not been evaluated. Literature review on brucellosis’ impact on women’s reproductive life found only one relevant reference. Needs further investigating.	[58–66]
Pollution with endocrine disrupters’ chemicals (EDC)	Regulations under-estimate health risks. Effects on reproductive life can appear at the next generation. Needs further investigating.	[67–73]
Human-Wildlife Conflict	Conflict arises from economic loss in agriculture, competition over food and water resources, fatalities in communities already ranking as the poorest in the world- No consideration of gendered impacts. Needs further investigating.	[74–77]
Poor access to natural resources and health care for Indigenous People, Local Communities, ethnic minorities	Poverty sustained by discriminatory processes, with women and girls bearing the burden of poverty and health care. Sense of “Biocultural dislocation” contributes to poor health status. Minority ethnic groups more severely affected by Covid-19 pandemic. IPLC’s rights need to be fully secured and their traditional knowledge preserved.	[78–84]
Food insecurity	One in 3 women affected by anemia. Perpetuates poverty cycles. Poorer access to services, technology, finances, land than men.	[23, 41, 85]
Climate change	Increased vulnerability and impacts associated with gender roles. Indigenous women particularly vulnerable through heavy reliance on natural resources and racial discrimination.	[41, 42, 86, 87]
**Women’s roles at the interface**	**Characteristics**	**References**
Agriculture	Share of women in agriculture under-estimated, increasing, mainly subsistence agriculture.	[23, 88–90]
Health care	Represent > 75% of health care workers. Both paid and unpaid. Great barriers to health education and services with gender norms. Economic weight perpetuates poverty cycle.	[91–94]
Plant biodiversity	Broad ethnobotanical knowledge and use of plant biodiversity. Wild plant harvesting used as a buffer from insecurity. Home gardening and seed selection increase resilience.	[95, 96]
Livestock	Poultry often the only livestock under women’s control, plays a critical role in poverty reduction and in vulnerable households. Role in selection and maintenance of breeds’ diversity. Greater exposure to EID’s risks.	[92–94, 97]
Other natural resources	Managing nearly all water-and energy-related aspects. Increased risks of injuries, sexual and physical violence.	[29, 41, 98]
**Examples of gender-sensitive and/or rights-based measures**	**Known outcomes**	**References**
Community-based governance systems for natural resources	Improved wealth and health of communities. Improved conservation of natural resources.	[76, 99, 100]
Recognition of indigenous management systems of natural resources	Improved conservation and health. Conservation of bio-cultural heritage.	[101]
Granting legal rights to ecosystems	Recognition of Indigenous People worldviews. New ways to protect ecosystems incl. transboundary ones	[102]
Vision of well- being of communities -*Buen Vivir*- in relation to their cultural and natural environment	Very strong environmental dimension with the Rights of Nature inscribed in the constitution.	[103]
State aspirational goals of good health, well-being, quality education	New models of societal progress with a better recognition of gender roles.	[104]
Women as head of state	Common features cited as resilience, pragmatism, benevolence, trust in collective common sense, mutual aid and humility Cited as better managers of the COVID-19 pandemic.	[105]
Presence of women law makers	Advances in gender equality, education and health care issues incl. Sexual and reproductive health, environmental issues, access to new economic opportunities.	[24, 106–108]
Gender equity in businesses	Improve business outcomes, creativity and innovation, attracts talents. Women more prone to social businesses.	[109]
Social and economic empowerment at household level	Improved health, education and quality of life of families and society. Breaks poverty cycles. Consolidate women as agents of deep transformative change (more concerned with environmental and climate issues than men).	[42, 87, 108]

### Conserving biodiversity needs a holistic approach - we need to learn from indigenous people and local communities (IPLCs)

Since the Convention on Biological Diversity in 1992, international commitments to halt biodiversity loss have been upscaled with the 2020 Aichi Biodiversity Targets and the 2030 Agenda for Sustainable Development. Main conservation policies implemented so far have largely focused on creating conservation areas that limit human use and access to such areas. In fact, Protected Areas coverage is the main conservation indicator used at national level. Some notable progress has at least been documented, with 15% of terrestrial and freshwater environments, and 6.3% of the world’s oceans now being protected [4]. But in face of mounting evidence showing nature’s dramatic decline, it is clear that the reality on the ground is a lot more complex and that many conservation initiatives have failed to be sustainable. The main drivers include land use changes associated with our consumption patterns and overexploitation of species, but a critical issue is the lack of recognition that Indigenous People and Local Communities^1^ (IPLCs) are the cornerstone of biodiversity and wildlands conservation.

Indigenous people own, occupy or manage land which holds 80% of the planet’s biodiversity and intersects with about 40% of all terrestrial protected areas and ecologically intact landscapes [110]. Biodiversity conservation therefore cannot be dissociated from the stewardship of indigenous people over some of the world’s most diverse and valuable natural resources. At least 370 million people define themselves as indigenous, based on an understanding of self-identification as such, of a historically distinct culture dating back to pre-colonial times, the use of distinct languages, and a strong link to surrounding natural resources [111]. Their vital role as managers and guardians of the world’s biodiversity is starting to be recognized, but will only be fulfilled if their land rights and self-determination can be secured, respected and supported [78]. Being the original inhabitants of a given regions, IPLCs should have the right to be considered as the true custodians of biodiversity, but in reality they are deprived from most of their land rights to access resources [4]. Indigenous people constitute around one-third of the world’s extremely rural poor people [111], and bear many of the costs associated with living conditions and risks emerging at the Human-Animal-Environment interface.

#### Costs of living at the interface

##### Emerging infectious diseases (EIDs)

A greater exposure to pathogens with a growing risk of disease transmission between humans, domestic animals and wildlife, contributes to poor health status and increased poverty of local communities, and to the risk of global pandemics of major importance, as currently experienced with SARS-CoV-2. Some human EIDs originate from infection with natural microbes, which cause no ill health in their wildlife hosts. However, when they spillover they may infect humans directly (Marburg), or via other animal species (HIV, avian flu, MERS-CoV), through genetic modification, amplification and increased virulence. Their emergence involves dynamic interactions among populations of wildlife, livestock and people, driven by human induced changes in the environment and degradation of ecosystems [50, 51]. While details of the underlying mechanisms and drivers of EIDs remains unclear, zoonotic EIDs are more likely to occur in regions with higher human and domestic animal populations densities and greater wildlife diversity, especially in fragmented tropical forests and wildlife markets [37, 52, 53].

The multiple gender dimensions of some EIDs, such as Ebola and HIV, are now acknowledged but EIDs are fairly recent diseases, with evolving epidemiology, which complicates the understanding of sex and gender-related risks factors. Gender analytical frameworks have been suggested to investigate the influence of sex and gender on vulnerability, exposure, response to infection and to public health interventions, but their implementation is still lagging behind, contributing to the maintenance of gender inequities and poverty cycles, thereby making disease eradication more difficult. With Ebola, gender-based differences in exposure occur at different stages of the outbreak in relation to gender specific activities. It appears that men in contact with infected primates, often hunters, became infected at the onset of the outbreak, but as the outbreak progressed, more women are exposed, due to specific roles as health carers (either at home or in the health center), as traditional healers, as midwives and as people who prepare the body for burial [47, 54]. There are also various sex-based differences associated with biological factors and women’s reproductive life stages, with pregnancy being a critical phase of increased vulnerability. Pregnant women infected with Ebola are at increased risk of abortion and neo-natal mortality whilst zika infection during pregnancy can cause a wide range of foetal abnormalities [55, 56]. Lassa fever, an EID linked to agriculturalisation of the environment, has a high case fatality rate in pregnancy, and is an important (and likely underreported) cause of maternal deaths in Lassa Fever-endemic areas in West African countries [57].

With SARS-CoV-2 it appears that, due to sex and gender differences, men and women are affected differently. Preliminary data analysis indicates there may be higher death rates in men than women, although data is limited to relatively few countries [112]. Sex related factors are likely to be involved as differences in immune systems between men and women are known to contribute to infectious disease response [113]. Gender related differences associated with risky behaviors (smoking, drinking) and co-morbidity including hypertension, cardiovascular disease and some chronic lung diseases might also be associated with adverse outcomes in cases with Covid-19 [114].

##### Endemic zoonotic diseases (ZDs)

Gender issues for more endemic ZDs such as brucellosis are yet to be integrated in One Health frameworks. There are increased risks of Neglected Zoonotic Diseases (NZDs) in low resource settings such as those surrounding conservation areas, causing significant morbidity and mortality and contributing to the burden of poverty. Brucellosis is the most widespread ZD in the world and is considered as an occupational disease for veterinarians, farmers and other professions handling animal products like milk and meat. It can be a major cause of abortions in cattle and is also known to affect pregnant women, but gender issues are only very rarely, if at all, examined. As part of this review, we conducted a reference search on PubMed for brucellosis publications between 2001 and 2018. Out of 14,304 publications found, only 1.5% pertained to health consequences of brucellosis on women’s reproductive life, which included abortions, premature delivery, intrauterine death, congenital brucellosis, misscarriages, neonatal brucellosis [58–65]. A preliminary review of these publications only detected one instance where gender issues rather than sex-related health consequences where examined: “*One distinct characteristic of the disease in this country is that the most cases are noted in female patients, by contrast to the rest of the world possibly reflecting an increased transmission as a foodborne disease (*via *milk cosumption) or increased participation of women in procedures associated with brucellosis transmission (eg. milking*)“ [66].

##### Pollution

Chemicals from pollution that can interfere with the normal development of human and wildlife, including endocrine disrupters’ chemicals (EDC) have been under scrutiny in the last decade. Examples of EDC are pharmaceutical estrogens, e.g. diethylstilbestrol (DES), polychlorinated biphenyls (used in the plastic industry), some pesticides, fungicides, phthalates, and also some naturally occurring ones, such as phytoestrogens. Recent results of the EU funded project EDC-MixRisk (2015–2019) indicate that current regulation of man-made chemicals, systematically underestimate health risks associated with combined exposures to EDC [67]. The effects on human and wildlife may occur long after the exposure, e.g. exposure to the fetus in the womb and effect in the reproductive apparatus of the developed adult or in future generations [68]. Gender issues have been brought into consideration in the EDC debate [69, 70] but need further investigation considering their impacts in early pregnancy, fetal development and genital malformations both in humans [71, 72] and in wildlife [73].

##### Human-wildlife conflict (HWC)

A great social cost borne by Indigenous People and Local Communities is a widespread Human-Wildlife Conflict, which will only increase as human populations grow, wildlife habitats shrink and competition over access to resources increases [74]. Conflicts generally arise from economic losses to agriculture, including loss of livestock through predation and destruction of crops, but also from competition over access to water and other resources, all of which contribute to food insecurity, increased psychological stress and sometimes human death [74]. In India at least one human life is lost every day due to conflict with elephants and tigers, which is proportionately smaller compared to lives lost from air pollution or car accidents, but it leads to growing disapproval of conservation measures and retaliatory injury or killing of animals [74]. The strong relationship that exists between elephant poaching rates in conservation areas and the poverty levels of communities living around conservation sites, illustrates the perversity of conservation strategies which fail to address development issues in communities which already rank among the poorest of the globe [75].

The need for a more holistic approach to conservation with more equal benefit sharing has been promoted for some time through pioneering community-based conservation schemes such as Campfire in Zimbabwe [99]. The positive impacts of conservation on local people’s wealth and health are most notable when there are community-based conservation areas or multiple-use Protected Areas allowing some form of resource use with community-based governance systems [100]. However, gender issues have been ignored when addressing HWC or Community Based Natural Resource Management (CBNRM) systems, despite the fact that women bear many of the hidden costs associated with current conservation strategies. This is associated with the gender division of labor (see next section), but also the invisible costs of HWC in term of time, workload, nutritional status and safety, which are mainly borne by women and which easily go unnoticed by wildlife authorities and researchers [76, 77].

##### Biocultural dislocation

The dramatic health status of many Indigenous Peoples is a product of social and cultural exclusion, environmental degradation and contamination of ecosystems in which they live, combined with a decline in access to traditional food and health systems. Indigenous People can be up to 60% poorer compared to benchmark populations in the same countries and rates of malnutrition in indigenous children can be double that of non-indigenous populations in Latin America [79]. Separation from a sense of place and identity associated with natural landscapes and biodiversity can create a sense of “biocultural dislocation” [80], which has profound negative social, health and psychological impacts on communities, through loss of access to agrobiodiversity resources, social and culinary traditions, and separation from traditional approaches to health care. Indigenous peoples are even more vulnerable now with the COVID-19 pandemic, owing to their lack of adequate health and social services, racial discrimination and significantly higher rates of non-communicable diseases [81].

##### A human rights issue

More broadly, access to natural resources (including clean air, clean energy, drinking water and natural food products), and to health care should be treated as human rights issues, which are central to the One Health concept. The depth, extent and consequences of racial inequities in the most affluent economies have now been fully exposed through the COVID-19 pandemic. In the UK, minority ethnic groups are reported to be to be disproportionately and more severely affected by COVID-19 than the country’s white majority. Patients from Black, Asian and Minority Ethnic (BAME) communities were found to account for 34% of critically ill COVID-19 patients nationally, despite constituting 14% of the UK’s population [82]. Overall deprivation was associated with worse coronavirus outcomes, and a more detailed examination of the living environment revealed that air pollution was also positively associated with an increase in COVID-19 cases and deaths, in the UK and the Netherlands [83, 84]. Of concern are the repercussions that the COVID-19 pandemic has already had on access to sexual and reproductive health and rights, which are fundamental to people’s health and survival.

Water security is also a basic human right. Access to potable water for sanitation and hygiene forms the basis of disease prevention (exemplified by COVID-19’s first line of defense measures), including food-, water- and vector-borne diseases [34]. However, 2.2 billion people are still living without access to safe drinking water and climate change will aggravate water stress for the most vulnerable ones [115]. Fair access to nutritious and balanced natural food should also be recognized as a human right since it is central to improved health and wellbeing of the world’s population and better stewardship of the planet [21].

### Caring about nature and health: gender roles and responsibilities are associated with unequal burdens and access to opportunities

The concept of gender refers to the social and cultural roles of males and females within a given society, which are shaped by cultural and social factors. Sex is defined as the biological characteristics that define men and women [116]. Gender roles and responsibilities within a family or society determine women’s workload and their engagement in productive and reproductive activities, as well as their vulnerability to health risks, biodiversity loss and climate change.

#### Caring for food security and family health

Traditionally societies have been divided along a male / female axis: those defined as female being usually allocated primary responsibilities for domestic labor and care of others in the family, while males are more identified with waged work and the duties of citizenship [88]. Women were associated with what is socially defined as the “domestic” realm, or “reproductive” sphere, where women carry out unpaid, home-based activities that ensure the maintenance and functioning of people within households and contribute to the perpetuation of humanity. In all but the most highly industrialized regions of the world, the “reproductive” sphere occupied by women is in reality a tremendously productive realm, contributing to the majority of subsistence resources in many rural areas [89]. Women in many LMICs continue to stay in rural areas and work in agriculture, and as the impacts of conflicts, HIV/AIDS and migration increase, the female share of the agricultural labor force rises and contributes to the agriculture feminization trend [90]. It is estimated that women comprise an average of 48% of the agricultural labor force, but their contribution is under-estimated, as they are less likely to define their activities (e.g. growing crops for food consumption) as work, whereas men tend to generate income from agricultural product sales [117].

Despite women’s key role in food systems and as the main person responsible for household food preparation, on which 85–90% of the time is spent, women tend to be more food-insecure than men. In Africa, more than a quarter of women above 15 years of age are experiencing severe food insecurity, meaning they go for entire days without eating, due to the lack of money or other resources [23]. In rural areas of LMICs, women’s heavy reliance on the goods and services provided by nature for their subsistence and livelihood makes them disproportionately affected by the loss of natural resources and therefore less resilient to environmental changes. As resources become scarcer, women are often the first to go hungry, or they might have to choose cheaper and less-healthy food that can lead to overweight and obesity. Anemia, an indicator of both poor nutrition and health and which affects economic and social development, currently affects one in three women of reproductive age worldwide, and shows an increasing prevalence [23]. During pregnancy, anemia increases risks of perinatal and maternal mortality, low birth weight and poor child growth and development from which long term poverty will emerge [41]. Malnutrition in women therefore contributes to perpetuating poverty cycles. Yet women as agricultural producers face greater constraints than their male counterparts in accessing essential productive resources and services, technology, market information and financial assets, while they are under-represented in local institutions and governance mechanisms, and tend to have less decision-making power [97].

In addition, women are more exposed to health risks of infectious diseases, in their roles as informal carers for the health of children and other family members, and in formal roles as paid health-care workers both at community and health institution levels. Not surprisingly, there have been more than twice as many cases of COVID-19 among female health-care workers in Spain compared to their male counterparts [95]. Women also often face greater barriers to health information and services, due to cultural gender norms such as economic dependence, patriarchal structures and a greater burden of domestic chores [118]. They also assume the emotional weight of caring for the sick, often without any psychological support, as well as an economic weight, as they sacrifice their education and careers to this role, perpetuating a vicious poverty circle associated with poor education and poor health [41]. The inconspicuous nature of the free labor of women in informal care roles makes them even more invisible [96]. It is estimated that women today contribute annually US$3 trillion to global health care, half of which is in the form of unpaid care work [85].

#### Caring for plant biodiversity

Wild plant harvesting plays an increasingly important role in food security. They provide highly nutritious food during the cultivated crop growing season, and at times of uncertainty during unpredictable or extreme weather events. However, these traditional food systems and gender roles have been overlooked, as modern market economies have promoted consolidated agriculture, based on few selected grains, producing cash crops for foreign markets, which are usually grown by men and in the process side-line women [117]. Until recently, research on household food security failed to have a gender-sensitive approach and to mention the contribution of home gardening or wild plant use that arises from women’s traditional knowledge [89].

As primary users of the natural resource base, women have a profound understanding of their environment and a broad knowledge about biodiversity, containing many insights into local species and ecosystems, gained from centuries of practical experience. Over much of the world, it is mainly women who are managing plant biodiversity, by managing the interface between wild and domesticated edible plant species. They are wild plant gatherers and managers, home gardeners and plant domesticators, herbalists and healers and are custodians of the whole seed cycle from selection, cleaning, storage to identifying which seeds to plant each season [89]. They use a wide range of criteria such as drought resistance, nutrition, taste, cooking time and storability when they select their seed. Their ethnobotanical knowledge allows to maintain the integrity of nutritional food through specific post-harvest processes such as detoxification or preservation of wild plant foods [89]. Through continuous use and seed exchange, women maintain the best genetic potential in their crops for dealing with environmental stresses, pests and diseases, as well as qualities, such as climate resilience, which are now paramount to future global food security.

#### Caring for livestock

Women play a prominent role in managing poultry and other small livestock that are housed and fed within the homestead, in contrast to the role men have in keeping and marketing larger livestock [97]. In many LMICs, poultry are often the only livestock under the independent control of women and also the most numerous livestock, contributing as much as 70% of total protein production [91].

By owning these village poultry productions systems, women engage in a relatively easy livestock management activity that contributes to household food security and income, and which is critical in poverty alleviation [92]. Since women are the main caretakers of family health, these production systems play an important role in vulnerable households such as female-headed ones and households affected by HIV/AIDS, by providing women with a source of income to carry out their tasks of supporting people [93].

Women as managers of small livestock play an important role in the maintenance and selection of the most adapted breeds to their changing environment. Village chicken of indigenous species have many advantages in comparison to commercial breeds as they require less veterinary inputs, scavenge for food and are more likely to survive in harsh environments [93]. With increasing climate variability and increasing incidence of illness in livestock, maintaining a healthy and diverse genetic reservoir in food-producing animals is now crucial for future food security [92].

However, by managing small livestock and relying on it for their livelihoods, women are exposed to greater risks, whilst also playing a key role in the epidemiology and prevention of zoonoses [94]. The loss of their precious poultry assets through Newcastle disease or avian influenza was found to deprive women from their source of income and to affect also the whole family poverty level and food security [47].

#### Caring for other natural resources

Women intersect with every aspect of water, including its access, management and water-related health risks in households that lack access to a potable water source. In 8 out of 10 households with an off-premises water source, women and girls are responsible for water collection [98]. On average, women in sub-Saharan Africa spend 18 h a week gathering fuel and water, compared to 5 h a week for those using clean fuels. Time spent on water-related activities deprives women from accessing education, earning an income and other critical livelihood activities that support safe, productive and healthy lives [28]. Collecting and carrying water while pregnant can also lead to reproductive disorders. In an increasingly water-stressed world where risks of political instability or conflict over water are growing, water management activities need to be gender-sensitive throughout. Women also bear the largest health burden from related fuel-gathering tasks, which are associated with increased risks of injuries, animal attacks and threats of physical or sexual violence [29] similar to risks associated with water collection tasks.

Climate change has gender-differentiated impacts as the skewed power relations and inequitable social norms often increase women’s vulnerability to these events. Overall, it is estimated that women and children are 14 times more likely to die during natural disasters than men [41]. Explanations for this include women being often less physically able than men to deal with such situations, and their tendency to prioritize the safety of children and household valuables, even in the event of a warning being issued, often waiting for the return of their relatives before they retreat to safety [86]. However, there are also indirect impacts of climate change on women’s lives which need to be considered, such as agricultural productivity decline or biodiversity loss due to droughts and post-disaster gender-based violence [87]. Indigenous women have been identified as particularly vulnerable to climate change impacts, primarily due to their reliance on natural resources for their livelihoods and the multiple discriminations they face due to their gender, ethnicity and level of poverty [42].

### A gender-responsive and rights-based One Health framework for healing nature and ourselves

Sustainable development strategies must value the roles of women, and that of indigenous and local communities, and their contributions to society and nature, their rights to healthy lives, and their rights to make their own decisions. Without so, a shift to more peaceful, inclusive and healthier societies, with sustainable economic paths and adaptive processes to build resilience, will remain no more than distant goals. The added benefits of adopting an integrated One Health approach to improve health and foster sustainable development for the Agenda 2030 have been demonstrated [34]. Gender equality is known to be fundamental to delivering on the promise of the same Agenda, which holds the potential of leaving no one behind and to protect and fulfil the rights of all [41].

**Fig. 1:**
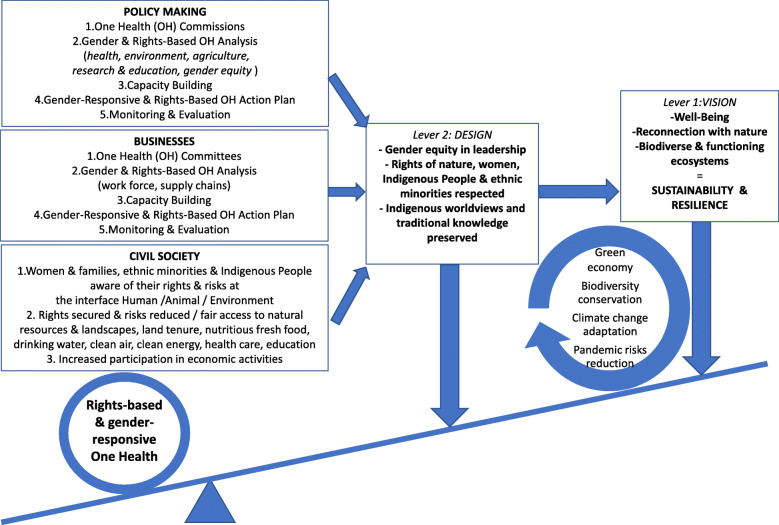
A framework for mainstreaming gender-responsive and rights-based One Health to deliver improved well-being for all and healing of nature. The framework uses a leverage points perspective for sustainability in complex systems [119, 120]. We suggest that the two most important leverage points to help heal nature and ourselves are: 1. A change of mindset and paradigm, going from a pursuit of wealth, GDP and overconsumption, towards a goal of well-being of humans and their re-connection to healthy and diverse ecosystems, using a holistic One Health understanding of health and nature. This would build resilience in the face of climate change and risks of future pandemics. We recommend learning from Indigenous People to re-position ourselves within nature and better conserve biodiversity. 2. The integration of gender equity in leadership and the respects of the rights of nature, women and the most vulnerable, including minority ethnic groups and Indigenous People. This leverage point requires actions at all levels (boxes on the left): Implementing a gender-responsive and rights-based One Health Action Plans in policy-making institutions and businesses, as well as a fair access to natural resources and landscapes, clean air, water and energy, nutritious fresh food, health care, land tenure and economic opportunities for women, ethnic minorities and Indigenous people by securing their rights. Action on these two levers would greatly contribute to developing a Green Economy, to conserving and restoring biodiversity and adapting to climate change, and to reducing risks of future pandemics (see description in the body text and supporting evidence in Table 1)

Our framework is based on a leverage points perspective as originally developed by Meadows [119], one of the world’s pioneers in research on sustainability in coupled human-environment systems. This perspective is best suited for finding the most important leverage points in complex systems, such as the socio-ecological and economic ones involved in the One Health approach. Meadows differentiated deep leverage points, where interventions are difficult but bring about transformative change, from shallow ones where interventions are easy, but limited in their potential to bring about transformative change. Following a simplification of deep leverage points into two realms (Intent e.g. goals of a system, paradigm etc.; and Design e.g. rules, powers to change system structure, structures of information flows) [120], we suggest that the two most important leverage points to help heal ourselves and nature are (Fig. 1):

- A change of mind-set and paradigm, from a pursuit of GDP and overconsumption, towards the well-being of humans and their reconnection to healthy environments using a holistic One Health understanding of nature and health. We recommend learning from Indigenous People and preserving traditional knowledge to re-position ourselves within nature and better conserve biodiversity.
- The integration of a gender-responsive and rights-based One Health approach at all levels of society, from policy-making to businesses and the civil society, leading to gender equity and the respect of the rights of nature, of women and of ethnic minorities.

#### From overconsumption towards well-being and re-connection to healthy environments

The pursuit of financial wealth by countries and corporate businesses is one of the main drivers behind the current societal and environmental challenges that include inequity, over-consumption, climate change and biodiversity loss. Such wealth systems have shown their limitations, and alternatives are needed, such as the global “beyond GDP” movement which has emerged. With aspirational goals like “good health and well-being” and “quality education” as measures of progress, some countries like New Zealand, Wales, Scotland and others in Latin America are developing models of societal progress where equitable and sustainable well-being is government’s ultimate goal [104]. The Organization for Economic Co-operation and Development (OECD) has constructed a Better Life Index*,* containing a range of metrics that better reflect what constitutes and leads to wellbeing, including trust, security, equality and sustainably [121]. While these new models represent a major step forward, they also need to integrate a reframing of our position within nature.

We need to learn from Indigenous People and Local people to re-position ourselves within nature and better conserve biodiversity***.*** Indigenous People acknowledge that their health and well-being, way of life, values and belief systems are entirely dependent on their respectful and holistic relationship they have with nature. Their culture, traditions and their artistic expressions are also a reflection of this complex and intimate relationship. Their worldview is one of being within nature rather than separation between humanity and nature, one of integration and inter-connectedness of all living forms on earth and the recognition that this inter-connectedness equates to a moral responsibility to care for, live in harmony with, and respect the natural world [122]. Nature is often given the status of a person, most often female, such as Pacha Mamma in South America or Mother Earth in First Nations. Recently, some ecosystems were granted legal personhood, such as the Whanganui River in New Zealand or the Ganges and Yamuna rivers in the state of Uttarakhand in India, in order to re-empower indigenous people to protect their ecosystems [102].

Indigenous management systems of natural resources, based on traditional indigenous knowledge and a holistic understanding of nature, have delivered remarkable conservation outcomes by sustaining and protecting genetic, species and ecosystem diversity [101]. Although there are dangers of reifying indigenous knowledge as an answer to all the world’s environmental problems, harnessing traditional environmental knowledge for biodiversity conservation and climate change adaptation is now increasingly recognized [123]. The relevance of indigenous knowledge systems, which are holistic and dependent upon relationships between all living beings cannot be ignored at a critical time when we need to re-balance ecosystems, health systems and food systems by implementing a One Health, system-thinking approach. The aboriginal concept of Oneness, meaning health and nature being one, epitomizes how we need to learn from those who still have a holistic understanding and deep respect for nature.

The *Buen Vivir* movement developed in Ecuador and Bolivia has a vision which focuses on the wellbeing of people within their community in relation to their specific cultural-natural environment and promotes the harmonious coexistence between mankind and nature [103]. It incorporates a strong environmental dimension as the Rights of Nature are actually inscribed in their constitutions. By re-constructing a system of knowledge and living, based on elimination of the Nature-Society dualism and the necessary interrelation of beings and knowledges, this new paradigm offers an alternative to the classical Western concept of development, based on the individual pursuit of prosperity and success [103]. The *Buen Vivir* does not provide a replicable model to all societies but rather it should be seen as a process in continual evolution which offers new answers to post-development challenges and which needs to be built in each specific context.

#### Mainstreaming gender-responsive and human-rights-based One Health

Mainstreaming a gender-responsive and human-rights-based One Health approach to protecting nature, improving health and well-being and adapting to climate change, would create an opportunity to influence the future direction of societies and help to overcome the multiple and inter-connected challenges faced by humanity today. By One Health we mean a truly holistic and respectful approach which re-position humans as one element of nature and re-establishes connections between these elements rather than the anthropocentric, arrogant and siloed view of nature, which has contributed to the biodiversity, climate, health and economic crises we are now facing. Gender-responsive implies processes and actions to overcome gender biases. In order to achieve this, we suggest that such mainstreaming needs to be undertaken at all levels of societies (Fig. 1), from government levels to private sector and civil society, after having removed gender and social discrimination.

In order to mainstream a gender-responsive and rights-based One Health approach at all levels of society, we advocate the creation of gender equitable One Health commissions and committees in both policy-making institutions and businesses respectively. The first step for these commissions is to examine the risks, vulnerability, obstacles faced by women and the most vulnerable, in terms of risks at the interface Human/Animal/Environment, the respect of human rights and a fair access to health care, education, leadership and economic opportunities, and to natural resources and natural landscapes. In businesses, the One Health committees will examine similar risks, obstacles and opportunities in the workforce and along supply chains, using a comprehensive Gender and Rights-Based Analysis [42, 124] . Following capacity building on the integration of gender equity and human rights in One Health within government and businesses, gender-responsive and rights-based One Health Action Plans will be developed and their progress will be monitored closely through adequate monitoring and evaluation. A diversity of outcomes can be expected at all levels as described below and summarized in Table 1.

##### Policy-making level

Despite some progress in bridging the gender gap, there are still major inequities in political and governance systems, which fail to address gender issues and to ensure that policies deliver equal outcomes for men and women. Globally, there are only 22 women in ministerial and parliamentary positions for every 100 men [106]. Although working for gender equality is obviously the responsibility of both male and female policy-makers, women’s participation and leadership in decision-making processes is known to lead to policy reforms that advance gender equality and the rights of women and girls [107]. The impact of women politicians’ leadership on health, environmental and climate outcomes is well documented. In the U.S., women lawmakers are more likely than men to sponsor bills related to education, health care and children issues [42, 108]. Correlations have been established between women in positions of political authority and lower carbon footprints as well as higher ratification of environmental treaties [42]. When included in decision-making relating to societal investment and resource use, women more often than men make decisions based on the best interests of children, family and community.

It might therefore not come as a surprise that under the current pandemic context, the actions of female leaders in countries such as Denmark, Germany and New Zealand were cited as supporting evidence that women were managing the crisis better than their male counterparts. Resilience, pragmatism, benevolence, trust in collective common sense, mutual aid and humility were mentioned as common features of the success of such women leaders. But these countries also rank high on the Global Gender Gap Report, meaning that the presence of female leadership is a reflection of more egalitarian societies [105].

The health sector is rightly described as being “delivered by women and led by men” and is one of the worse affected by gender inequality, as revealed by the current pandemic [125, 126]. Women make up 70% of the health workforce, but only 25% of global health organisations have gender parity at senior management levels [85]. In addition, the average gender pay gap is around 28% in this sector, with occupational segregation and harassment resulting in the weakening of health systems and the delivery of health services [85]. There is no doubt that health systems will be stronger when the women who deliver them have an equal say in the design of national health plans, policies and systems, especially with issues such as Sexual, Reproductive, Maternal, Newborn, Child and Adolescent Health policies, where gender considerations are often reduced to the collection of sex disaggregated data [108].

In the conservation domain a few women now hold top positions, but traditionally this workplace has been dominated by a few voices, nearly all of them white and male, reflecting the bigger issues of gender and cultural bias that have contributed to hinder the sustainability of conservation strategies [127]. Involving indigenous people and especially women’s voices will not only ensure the integration of their perspectives and knowledge in policy-making and implementation but it would ensure that the design of interventions fits with the reality of the situation on the ground - something that has so often been lacking.

The lack of access to leadership levels is one of the 10 “impact zones” that the McKinsey Global Institute identified as reflecting the seriousness of gender inequality globally and where targeted action could move women closer to parity [106]. The three biggest impact zones identified to affect women globally are fewer legal rights (affecting 2.5 billion women), time spent in unpaid care work (1.3 billion women), and violence against women (723 million). Other impact zones include low maternal and reproductive health, low labor-force participation in quality jobs, unequal education levels, financial and digital exclusion, and girl-child vulnerability, which are in fact inter-related. While the World Economic Forum estimates that it will take 81 years to close the gender gap completely, the McKinsey Global Institute identified a set of interventions that can accelerate gender gap closure. Among them, laws and policies, together with education and training, are considered to be the bedrock of efforts to open doors to new businesses. Investment in public infrastructures such as clean running water, sanitation and more effective cooking fuel are also key, allowing to reduce the amount of time women spend on household chores and therefore allowing them to devote more time to education and income-generating activities.

##### Businesses

While governments and policy-making institutions certainly hold critical roles in shifting directions to sustainability, gender and social inclusion, partnership with the private sector, engaging multiple stakeholders, is also key to drive a paradigm shift towards rights-based One Health. The antagonistic role that businesses can have for the 2030 Agenda under current political economies and the negative impacts that extractive industries and agri-businesses have on health and nature are well acknowledged [4]. Promoting gender equity in businesses is not only important as a human-rights issue, but gender diversity has also been found to improve business outcomes, attract talent and improve business’ creativity and innovation [109]. Although there is progress in the representation of women in corporate businesses, parity remains out of reach for women, and particularly women of color, who are underrepresented at every level [106]. Interestingly, the gender gap has been found to decrease in social businesses which are directly related to care, altruism and protection of others, with more women starting a social business compared to a purely commercial business with the sole purpose of creating an economic benefit [128]. This can be attributed to women being typically closer to social issues in both their private and professional lives, as a consequence of traditional gender roles inscribed in societies.

##### Civil society

At the household level, empowering women to participate in decision-making is crucial to improve health and quality of life for women and for their families. Women with greater agency are more likely to access health services, including reproductive and maternal health, and to have fewer children that have higher survival rates, receive better childcare at home and receive health care when they need it [108]. Healthy women and girls can in turn participate more actively in society and take action to advance their own interests, but the essential first step is for women to be fully aware of their rights, including their rights to health, which include four inter-related elements: availability, accessibility (non-discriminatory and affordable), acceptability (respectful and gender-responsive) and quality [108]. The importance of economically empowering women in households, particularly poor ones headed by widows or where men are absent, is also known to greatly contribute to poverty reduction. When households have access to food, energy and particularly renewable energy, water and sanitation, then women can devote more time to income generating activities as well as education and leisure [87]. Another important area, where closing the gender gap would have a significant impact on poverty reduction, is agriculture and especially rights to land tenure, which could increase crop production by 2 to 7.3% and lift hundreds of thousands people out of poverty in many countries [87].

Promoting women as agents of change, to lead mitigation and adaptation efforts in the face of climate change, is also essential. Women generally are more concerned than men about climate change, they feel a greater need for action in tackling climate change and are more likely to change their behavior, while men have more trust in technological solutions [42]. In LMICs which produce fewer emissions and consume less than high income countries but which are the most affected by climate change, the integration of a gender perspective has led to many positive outcomes of projects aiming at empowering women economically, mitigating or adapting to climate change and managing natural resources sustainably [42].

Acknowledging that women at all levels of society and decision-making can be powerful agents of deep transformative change and giving them equal rights and opportunities, represents the keystone to seeing a reversal in the process that is currently threatening our civilization, as demonstrated by the COVID-19 pandemic. While a sense of urgency is undoubtedly growing in civil society, the divide between current political trajectories is also increasing, representing a major threat to the global commitment that is now required. We have a unique window of opportunity to “Build Back Better” now by creating more resilient, inclusive and sustainable societies [129], but this depends on our ability to conserve and restore biodiversity and re-connect with nature. As a wise old man in Africa once said, whilst facing a life threatening event, “I hope that I’ll be able to survive to see what happens next!”.

## Conclusion

The current biodiversity, health and climate crisis that humanity faces today has been driven by the old development paradigm, prioritizing the pursuit of wealth and food security, leading to the decline of biodiversity and ecosystem services that sustain life and good health. More than a century of conservation efforts have not managed to halt this dramatic environmental crisis. We argue that the integration of a gender perspective together with the vision, traditional knowledge and needs of Indigenous Peoples and Local communities, into a multi-sectoral One Health approach, would greatly enhance biodiversity conservation, global health and sustainable development outcomes. More particularly, by reviewing the roles and responsibilities that local and indigenous women hold in rural and more traditional societies, we highlight their pivotal role in managing and conserving natural resources, but also the disproportionate social costs and health burden that they bear in relation to men. By examining the multiple challenges that emerge at the Human-Animal-Environment interface, we found that the integration of a gender perspective has been initiated for some emerging infectious diseases and climate change issues, but that the implementation is lagging behind, as demonstrated during the COVID-19 pandemic. For other social costs, which include endemic ZDs (e.g. brucellosis), pollution, Human-Wildlife Conflict and the restricted access to natural resources, and that are borne by people living at the interface, the consideration of a gender perspective is mainly absent. The One Health approach provides a unique opportunity to address the multiple and inter-connected challenges we currently face, including risks of pandemics, biodiversity loss and climate change, but gender inequities are still prevalent at all levels of societies and particularly in the health sector. By illustrating how women can be agents of change in their communities, in the political domain or in the private sector, we suggest in a framework that mainstreaming a gender-responsive and rights-based One Health approach at all levels could help reverse the inter-dependent processes of environmental, health and climate degradation, and move towards building resilience of humanity and nature.

## Data Availability

Not applicable.

## Footnotes

1 Indigenous peoples and local communities (IPLCs) are, typically, ethnic groups who are descended from and identify with the original inhabitants of a given region, in contrast to groups that have settled, occupied or colonized the area more recently. According to the United Nations, a modern understanding of the term “indigenous” is also based on a strong link to surrounding natural resources, distinct languages and culture, a resolve to maintain their ancestral environments and systems as distinctive peoples.

## References

[1] Guterrez A. Secretary-General’s remarks at 2019 Climate Action Summit. https://www.un.org/sg/en/content/sg/statement/2019-09-23/secretary-generals-remarks-2019-climate-action-summit-bilingual-delivered-please-scroll-down-for-all-english-version. Accessed 23 Sept 2020.

[2] WHO (2020). Rolling updates on cornovirus disease (COVID-19).

[3] Cardinale BJ, Duffy JE, Gonzalez A (2012). Biodiversity loss and its impact on humanity. Nature..

[4] Ferrier S, Ninan KN, Leadley P, et al. Summary for policymakers of the methodological assessment of scenarios and models of biodiversity and ecosystem services of the intergovernmental science-policy platform on biodiversity and ecosystem services. Secr Intergov Sci Platf Biodivers Ecosyst Serv. 2019. 10.1590/1676-0611201600010001.

[5] Mace GM, Reyers B, Alkemade R (2014). Approaches to defining a planetary boundary for biodiversity. Glob Environ Chang.

[6] Ripple WJ, Wolf C, Newsome TM, Barnard P, Moomaw WR (2019). World Scientists’ Warning of a Climate Emergency. Bioscience.

[7] Myers SS (2017). Planetary health: protecting human health on a rapidly changing planet. Lancet..

[8] Pingali PL (2012). Green revolution: impacts, limits, andthe path ahead. Proc Natl Acad Sci U S A.

[9] You D, Hug L, Ejdemyr S (2015). Levels & Trends in Child Mortality.

[10] Díaz S, Settele J, Brondízio ES, et al. Pervasive human-driven decline of life on Earth points to the need for transformative change. Science. 2019;366(6471). 10.1126/science.aax3100. 10.1126/science.aax3100 31831642

[11] Whitmee S, Haines A, Beyrer C (2015). Safeguarding human health in the Anthropocene epoch: report of the Rockefeller Foundation-lancet commission on planetary health. Lancet..

[12] UNITED NATIONS (2019). World Population Prospects 2019: Highlights.

[13] World Resource Institute (2019). The new Aqueduct Water Risk Atlas: What’s New & Why Does It Matter to You?.

[14] Lebreton L, Slat B, Ferrari F (2018). Evidence that the great Pacific garbage patch is rapidly accumulating plastic. Sci Rep.

[15] European Commission (2019). Environmental and Health Risks of Microplastic Pollution.

[16] Bergmann M, Gutow L, Klages M. Marine anthropogenic litter. Mar Anthropog Litter. 2015:1–447. 10.1007/978-3-319-16510-3.

[17] Galloway TS, Cole M, Lewis C (2017). Interactions of microplastic debris throughout the marine ecosystem. Nat Ecol Evol.

[18] Ceballos G, Ehrlich PR, Dirzo R (2017). Biological annihilation via the ongoing sixth mass extinction signaled by vertebrate population losses and declines. Proc Natl Acad Sci U S A.

[19] Steffen W, Grinevald J, Crutzen P, Mcneill J (2011). The anthropocene: conceptual and historical perspectives. Philos Trans R Soc A Math Phys Eng Sci.

[20] Settele J, Diaz S, Brondizio EDP (2020). COVID-19 Stimulus Measures Must Save Lives, Protect Livelihoods, and Safeguard Nature to Reduce the Risk of Future Pandemics.

[21] Alders R, Cribb J, Nunn M, Kock R, Rushton J, Bagnol B. Approaches to Fixing Broken Food Systems. In: Eggersdorfer M, Kraemer K, Cordaro JB, Fanzo J, Gibney M, Kennedy E, Labrique A, editors. Good Nutrition: Perspectives for the 21st Century. 1st ed. Basel Karger; 2016.

[22] Alders RG, Ratanawongprasat N, Schönfeldt H, Stellmach D (2018). A planetary health approach to secure, safe, sustainable food systems: workshop report. Food Secur.

[23] FAO (2019). Food Security and Nutrition in the World.

[24] Sanyaolu A, Okorie C, Marinkovic A, Patidar R, Younis K, Desal P, Hosein Z, Padda I, Altaf M. Comorbidity and its impact on patients with COVID-19. SN Compr Clin Med. 2020. 10.1183/13993003.01227-2020. 10.1007/s42399-020-00363-4 PMC7314621 32838147

[25] Darnton-Hill I, Nishida C, James W (2004). A life course approach to diet, nutrition and the prevention of chronic diseases. Public Health Nutr.

[26] Ma RCW, Popkin BM (2017). Intergenerational diabetes and obesity—a cycle to break?. PLoS Med.

[27] WHO. Household air pollution and health. https://www.who.int/news-room/fact-sheets/detail/household-air-pollution-and-health. Accessed 23 Sept 2020.

[28] Austin KF, Mejia MT (2017). Household air pollution as a silent killer: women’s status and solid fuel use in developing nations. Popul Environ.

[29] WHO. Burning Opportunity: Burning Opportunity. World Heal Organ. 2016:1–113 https://www.afro.who.int/sites/default/files/2017-06/9789241565233_eng.pdf.

[30] Wallace RG, Kock RA (2012). Whose food footprint? Capitalism, Agriculture and the Environment. Hum Geogr.

[31] UNITED NATIONS (2019). No Time to Wait:Securing the Future from Drug-Resistant Infections.

[32] Robinson TP, Bu DP, Carrique-Mas J (2016). Antibiotic resistance is the quintessential one health issue. Trans R Soc Trop Med Hyg.

[33] WHO (2018). Tackling Antimicrobial Resistance (AMR) Together.

[34] Queenan K, Garnier J, Nielsen LR, et al. Roadmap to a One Health Agenda 2030. CAB Rev Perspect Agric Vet Sci Nutr Nat Resour. 2017;12(014). 10.1079/PAVSNNR201712014.

[35] Rüegg SR, Nielsen LR, Buttigieg SC (2018). A systems approach to evaluate One Health initiatives. Front Vet Sci.

[36] Halliday JEB, Allan KJ, Ekwem D, Cleaveland S, Kazwala RR, Crump JA (2015). One health: endemic zoonoses in the tropics: a public health problem hiding in plain sight. Vet Rec.

[37] UNEP & ILRI. Preventing the Newt Pandemic: Zoonotic Diseases and How to Break the Chain of Transmission https://wedocs.unep.org/bitstream/handle/20.500.11822/32316/ZP.pdf?sequence=1&isAllowed=y. Accessed 23 Sept 2020.

[38] Marty AM, Jones MK (2020). The novel coronavirus (SARS-CoV-2) is a one health issue. One Heal.

[39] One Health Institute UD (2020). PREDICT Project.

[40] UN (2019). The Sustainable Goals Development Report.

[41] UN Women (2018). Turning promises into action: gender equality in the 2030 agenda for Sustain Dev.

[42] UNFCCC (2019). Differentiated impacts of climate change on women and men, integration of gender considerations into climate policies, plans and actions, and progress in enhancing gender balance in national climate delegations.

[43] WHO (2019). Thirteenth General Programme of Work, 2019–2023: Promote health. Keep the world safe Serve the vulnerable.

[44] Wenham C, Smith J, Morgan R (2020). COVID-19: the gendered impacts of the outbreak. Lancet..

[45] FAO, OIE, WHO (2019). A Tripartite Guide to Addressing Zoonotic Diseases in Countries.

[46] Amuguni JH, Mugisha A, Kyewalabye E, et al. Adv Soc Sci Res J. 2018;5(5). 10.14738/assrj.55.4640.

[47] Bagnol B, Alders R, McConchie R. Gender Issues in Human, Animal and plant health using an Ecohealth Perspective. Environ Nat Resour Res. 2015;5(1). 10.5539/enrr.v5n1p62.

[48] Friedson-Ridenour S, Dutcher TV, Calderon C, Brown LDP, Olsen CW (2019). Gender analysis for one health: theoretical perspectives and recommendations for practice. Ecohealth..

[49] Rampa BF, Dekeyser K, Alders R, Dar O (2019). The global institutional landscape of food and agriculture: how to achieve SDG2.

[50] Jones KE, Patel NG, Levy MA (2008). Global trends in emerging infectious diseases. Nature..

[51] Karesh WB, Dobson A, Lloyd-Smith JO (2012). Ecology of zoonoses: natural and unnatural histories. Lancet.

[52] Allen T, Murray KA, Zambrana-Torrelio C, et al. Global hotspots and correlates of emerging zoonotic diseases. Nat Commun. 2017;8(1). 10.1038/s41467-017-00923-8. 10.1038/s41467-017-00923-8 PMC5654761 29066781

[53] Morse SS (1995). Factors in the emergence of infectious diseases. Emerg Infect Dis.

[54] Davies SE, Bennett B (2016). A gendered human rights analysis of Ebola and Zika: locating gender in global health emergencies. Int Aff.

[55] Bebell LM, Oduyebo T, Riley LE (2017). Ebola virus disease and pregnancy: a review of the current knowledge of Ebola virus pathogenesis, maternal, and neonatal outcomes. Birth Defects Res.

[56] Rasmussen SA, Jamieson DJ, Honein MA, Petersen LR (2016). Zika virus and birth defects — reviewing the evidence for causality. N Engl J Med.

[57] Okogbenin S, Okoeguale J, Akpede PG (2019). Retrospective cohort study of Lassa fever in pregnancy, southern Nigeria. Emerg Infect Dis.

[58] Kurdoglu M, Cetin O, ZAH K (2015). The effect of brucellosis on Women’s Health and Reproduction. Int J Women’s Heal Reprod Sci.

[59] Khan MY, Mah MW, Memish ZA (2001). Brucellosis in pregnant Women. Clin Infect Dis.

[60] Vilchez G, Espinoza M, D’Onadio G, Saona P, Gotuzzo E (2015). Brucellosis in pregnancy: clinical aspects and obstetric outcomes. Int J Infect Dis.

[61] Ali S, Akhter S, Neubauer H (2016). Brucellosis in pregnant women from Pakistan: an observational study. BMC Infect Dis.

[62] Ntirandekura JB, Matemba LE, Kimera SI, Muma JB, Karimuribo ED (2018). Association of brucellosis with abortion prevalence in humans and animals in Africa: A review. Afr J Reprod Health.

[63] Arenas-Gamboa AM, Rossetti CA, Chaki SP (2016). Human brucellosis and adverse pregnancy outcomes. Curr Trop Med Reports.

[64] Agah J. Different Manifestation of Brucellosis in Pregnancy: Case Reports. J Microbiol Infect Dis. 2016:80–3. 10.5799/ahinjs.02.2016.02.0221.

[65] Štrbac M, Ristić M, Petrović V (2017). Epidemiological characteristics of brucellosis in Vojvodina, Serbia, 2000-2014. Vojnosanit Pregl.

[66] Pappas G, Papadimitriou P, Akritidis N, Christou L, Tsianos EV. The new global map of human brucellosis. Lancet Infect Dis. 2006. 10.1016/S1473-3099(06)70382-6. 10.1016/S1473-3099(06)70382-6 16439329

[67] Gennings C, Shu H, Rudén C, et al. Incorporating regulatory guideline values in analysis of epidemiology data. Environ Int. 2018. 10.1016/j.envint.2018.08.039. 10.1016/j.envint.2018.08.039 PMC6261378 30170308

[68] Sifakis S, Androutsopoulos VP, Tsatsakis AM, Spandidos DA. Human exposure to endocrine disrupting chemicals: effects on the male and female reproductive systems. Environ Toxicol Pharmacol. 2017. 10.1016/j.etap.2017.02.024. 10.1016/j.etap.2017.02.024 28292651

[69] Hood E (2005). Are EDCs blurring issues of gender?. Environ Health Perspect.

[70] Langston N (2008). The retreat from precaution:regulating diethylstilbestrol (DES), endocrine disruptors, and environmental health. Environ Hist Durh N C.

[71] Roig B, Mnif W, Hadj Hassine AI (2013). Endocrine disrupting chemicals and human health risk assessment: a critical review. Crit Rev Environ Sci Technol.

[72] Kelley AS, Banker M, Goodrich JM (2019). Early pregnancy exposure to endocrine disrupting chemical mixtures are associated with inflammatory changes in maternal and neonatal circulation. Sci Rep.

[73] Tubbs CW, McDonough CE (2018). Reproductive impacts of endocrine-disrupting chemicals on wildlife species: implications for conservation of endangered species. Annu Rev Anim Biosci.

[74] Shaffer LJ, Khadka KK, Van Den Hoek J, Naithani KJ (2019). Human-elephant conflict: A review of current management strategies and future directions. Front Ecol Evol.

[75] Hauenstein S, Kshatriya M, Blanc J, Dormann CF, Beale CM. African elephant poaching rates correlate with local poverty, national corruption and global ivory price. Nat Commun. 2019;10(1). 10.1038/s41467-019-09993-2. 10.1038/s41467-019-09993-2 PMC6538616 31138804

[76] Ogra MV (2008). Human-wildlife conflict and gender in protected area borderlands: a case study of costs, perceptions, and vulnerabilities from Uttarakhand (Uttaranchal), India. Geoforum.

[77] Khumalo KE, Yung LA (2015). Women, human-wildlife conflict, and CBNRM: hidden impacts and vulnerabilities in Kwandu conservancy, Namibia. Conserv Soc.

[78] FAO (2015). FAO Policy on Indigenous and Tribal Peoples.

[79] Anderson I, Robson B, Connolly M (2016). Indigenous and tribal peoples’ health (the lancet–Lowitja Institute global collaboration): a population study. Lancet..

[80] CE K (2017). Bio-cultural diversity and human well-being.

[81] United Nations. COVID-19 and Indigenous peoples: United Nations; 2020. https://www.un.org/development/desa/indigenouspeoples/covid-19.html.

[82] Public Health England (2020). Beyond the Data : Understanding the Impact of COVID-19 on BAME Groups.

[83] Soltan M, Crowley L, Melville C. To what extent are social determinants of health, including household overcrowding, air pollution and housing quality deprivation, modulators of presentation, ITU admission and outcomes among patients with SARS-COV-2 infection in an urban catchment area i. *Res Sq*. 2020; https://www.researchgate.net/publication/342814465_To_what_extent_are_social_determinants_of_health_including_household_overcrowding_air_pollution_and_housing_quality_deprivation_modulators_of_presentation_ITU_admission_and_outcomes_among_patients_wit#ful. PMC7614405 37034137

[84] Cole MA, Ozgen C, Strobl E (2020). Air Pollution Exposure and COVID-19.

[85] Betron M, Bourgeault I, Manzoor M (2019). Time for gender-transformative change in the health workforce. Lancet..

[86] WHO (2014). Gender, Climate Change and Health.

[87] UNEP & IUCN (2019). Unlocking Information for Action and Measuring the SDGs.

[88] Doyal L (2016). Sex, gender and health: a preliminary conceptual framework. Tant qu’on a la santé.

[89] Howard P (2003). Women and plants*.* Gender Relations in Biodiversity Management and Conservation.

[90] FAO-ONU (2017). The Future of Food and Agriculture: Trends and Challenges.

[91] Wong JT, de Bruyn J, Bagnol B (2017). Small-scale poultry and food security in resource-poor settings: a review. Glob Food Sec.

[92] Pym R, Alders R (2016). Helping smallholders to improve poultry production.

[93] Alders RG, Pym RAE. Village poultry: still important to millions, eight thousand years after domestication. Worlds Poult Sci J. 2009. 10.1017/S0043933909000117.

[94] Alders R, Aongolo A, Bagnol B, et al. Planet@Risk Spec Issue One Heal. 2014;2(3) http://planet-risk.org/index.php/pr/article/view/67/203.

[95] UN Women (2020). COVID-19 and gender: What do we know; what do we need to know?.

[96] Harman S (2016). Ebola, gender and conspicuously invisible women in global health governance. Third World Q.

[97] Gender Equality; 2008. doi: 10.5840/wcp22200825655.

[98] WHO/UNICEF (2017). Progress on Drinking Water, Sanitation and Hygiene.

[99] Child B (2019). Sustainable governance of wildlife and community-based natural resource management.

[100] Naidoo R, Gerkey D, Hole D (2019). Evaluating the impacts of protected areas on human well-being across the developing world. Sci Adv.

[101] Agnoletti M (2006). Conservation of cultural landscapes.

[102] O’Donnell EL, Talbot-Jones J. Creating legal rights for rivers: Lessons from Australia, New Zealand, and India. Ecol Soc. 2018;23(1). 10.5751/ES-09854-230107.

[103] Gudynas E (2011). Buen Vivir: Today’s tomorrow. Development..

[104] Salvaris M, Stanley F, Lycett K (2019). It’s time to vote for happiness and well-being, not mere economic growth. Here’s why. Conversat.

[105] Champoux-Paillé L, Croteau A-M (2020). Why women leaders are excelling during the coronavirus pandemic.

[106] Gupta V, Hieronimus S, Krishnan M, Madgavkar A (2019). Accelerating gender parity: What can governments do?.

[107] GCF/UN Women (2017). Mainstreaming gender in Green Climate Fund projects.

[108] UN Women (2019). Promoting gender equality in sexual , Reproductive , Maternal , Newborn , Child. Programming Guide.

[109] International Labor Office-Geneva:ILO (2019). The Business Case for Change The Business Case for Change.

[110] Garnett S, Fernandez-Llamazares A, Robinson C, et al. Indigenous peoples are crucial for conservation - a quarter of all land is in their hands. Conversat. 2018; https://theconversation.com/indigenous-peoples-are-crucial-for-conservation-a-quarter-of-all-land-is-in-their-hands-99742.

[111] UN. State of the World’s Indigenous Peoples. New York; 2009. https://www.un.org/development/desa/indigenouspeoples/.

[112] World Health Organization (WHO) (2020). Gender and COVID-19.

[113] Klein SL, Flanagan KL (2016). Sex differences in immune responses. Nat Rev Immunol.

[114] Purdie A, Hawkes S, Buse K, et al. Sex, gender and COVID-19: disaggregated data and health disparities. Blog BMJ Glob Heal. 2020;2019 https://blogs.bmj.com/bmjgh/2020/03/24/sex-gender-and-covid-19-disaggregated-data-and-health-disparities/.

[115] UN Water (2020). Water and Climate Change - UN World Water Development Report 2020.

[116] Broughton DE, Brannigan RE, Omurtag KR (2017). Sex and gender: you should know the difference. Fertil Steril.

[117] Bertram GC (1967). The state of food and agriculture, 1966. Eugen Rev.

[118] Boniol M, McIsaac M, Xu L, Diallo K (2019). Gender Equity in the Health Workforce: Analysis of 104 Countries.

[119] Meadows D (1999). Leverage points: places to intervene in a system.

[120] Abson D, Fischer J, Leventon J (2017). Al. E. Leverage points for sustainability transformation. Ambio..

[121] Stigliz J, Fitoussi J, Durand M (2018). Beyond GDP*:* Measuring What Counts for Economic and Social Performance.

[122] Simpson L. In: Oakes J, Riew R, Koolage S, Simpson L, NS, editors. Anishinaabe Ways of Knowing. Winnipeg; 2000.

[123] Makondo CC, Thomas DSG. Climate change adaptation: linking indigenous knowledge with western science for effective adaptation. Environ Sci Pol. 2018. 10.1016/j.envsci.2018.06.014.

[124] Galano MM, Graham-Bermann SA. Gender Analysis. Wiley Blackwell Encycl Gend Sex Stud. 2016:1–3. 10.1002/9781118663219.wbegss057.

[125] EIGE (2020). Frontline Workers.

[126] World Health Organization (WHO) (2019). Summary: Delivered by Women, Led by Men: A Gender and Equity Analysis of the Global Health and Social Workforce - Executive summary. Hum Resour Heal Obs.

[127] Jones MS, Solomon J (2019). Challenges and supports for women conservation leaders. Conserv Sci Pract.

[128] Nicolás C, Rubio A (2016). Social enterprise: Gender gap and economic development. Eur J Manag Bus Econ.

[129] UN (2020). Climate Change and COVID-19: UN urges nations to ‘recover better’.

